# Active site diversification of P450cam with indole generates catalysts for benzylic oxidation reactions

**DOI:** 10.3762/bjoc.11.186

**Published:** 2015-09-22

**Authors:** Paul P Kelly, Anja Eichler, Susanne Herter, David C Kranz, Nicholas J Turner, Sabine L Flitsch

**Affiliations:** 1 School of Chemistry & Manchester Institute of Biotechnology, The University of Manchester, 131 Princess Street, M1 7DN, Manchester, United Kingdom

**Keywords:** active site mutagenesis, biotransformation, C–H activation, cytochrome P450cam monooxygenase, hydroxylation

## Abstract

Cytochrome P450 monooxygenases are useful biocatalysts for C–H activation, and there is a need to expand the range of these enzymes beyond what is naturally available. A panel of 93 variants of active self-sufficient P450cam[Tyr96Phe]-RhFRed fusion enzymes with a broad diversity in active site amino acids was developed by screening a large mutant library of 16,500 clones using a simple, highly sensitive colony-based colorimetric screen against indole. These mutants showed distinct fingerprints of activity not only when screened in oxidations of substituted indoles but also for unrelated oxidations such as benzylic hydroxylations.

## Introduction

Selective C–H activation and oxyfunctionalisation of hydrocarbons offers a route to chiral alcohols and other industrially important synthetic building blocks from low cost starting materials [1]. One of the most attractive reagents in terms of cost and environmental impact for hydrocarbon oxidation is oxygen in the presence of a catalyst. In this context enzymatic oxidations are attractive, in particular cytochrome P450 monooxygenases (P450s or CYPs) due to their ability to catalyse selective C–H bond oxidations under mild conditions [2].

The soluble bacterial camphor monooxygenase P450cam (CYP101A1, EC 1.14.15.1) from *Pseudomonas putida* is one of the most studied P450s and has been engineered to accept a variety of non-natural substrates including aryl–alkyl compounds [3], olefins [4], polycyclic aromatic hydrocarbons [5], terpenes [6–8] and alkanes as small as ethane [9]. Over the years a number of active site mutants of P450cam have been generated by rational re-design, but the active site has not been explored in a comprehensive and systematic manner. Given that P450cam is a robust biocatalyst with good activity for this class of enzymes, a library of active site mutants with diversity in amino acid side chains lining up the substrate pocket would demonstrate a valuable resource for the development of useful P450cam based biocatalysts.

The generation of libraries of active enzyme mutants requires efficient screening protocols, which is a particular challenge for P450s given that (i) a diverse range of oxidations are catalysed by the enzymes, generally without intrinsic change in chromophore; (ii) the potential substrate range and diversity is high; (iii) each substrate might result in many different oxidation products. Here, we describe how such issues can be overcome by (i) using surrogate high-throughput screening (HTS) protocols that can deal with a large number of mutants; (ii) identification of active mutant libraries; (iii) fingerprinting of these libraries against substrates for a broad substrate panel, activity and chemo-, regio- and stereoselectivity.

The production of indigo from indole derivatives **1**–**4** by P450s can be considered as an effective visual screen for identifying interesting new mutants from diverse libraries. Indole hydroxylation by P450cam [10–11] and various other P450s, including the bacterial P450 BM3 [12–13] and human CYPs 2A6, 2C19 and 2E1 [14–15] has been previously identified to translate well to mutants activities toward structurally distinct and more demanding substrates such as diphenylmethane [10], phenacetin, ethoxyresofurin and chlorzoxazone to only name a few [16].

For the current investigation we sought to develop P450cam further to expand their substrate range in biocatalysis. Our starting point was a catalytically self-sufficient form of the enzyme, previously created by fusion with the reductase domain of P450-RhF (RhFRed) [17–19]. This chimera, named P450cam-RhFRed, operates without the need for additional reductase partners and retains the native activity of non-fused P450cam in the selective oxidation of camphor to 5-*exo*-hydroxycamphor. When generated as a whole-cell biocatalyst in *Escherichia coli* (*E*. *coli*), variants of the fusion enzyme catalysed the efficient, highly selective hydroxylation of ionones without the need to supply expensive nicotinamide cofactors [20]. Given the previously demonstrated affinity of P450cam for hydrophobic substrates, we were interested to see if P450cam-RhFRed could be used as a template for engineering variants for the stereoselective benzylic hydroxylation of substituted aromatics **5**–**8**.

## Results and Discussion

### Introducing structural and functional diversity into P450cam

**Figure 1:**
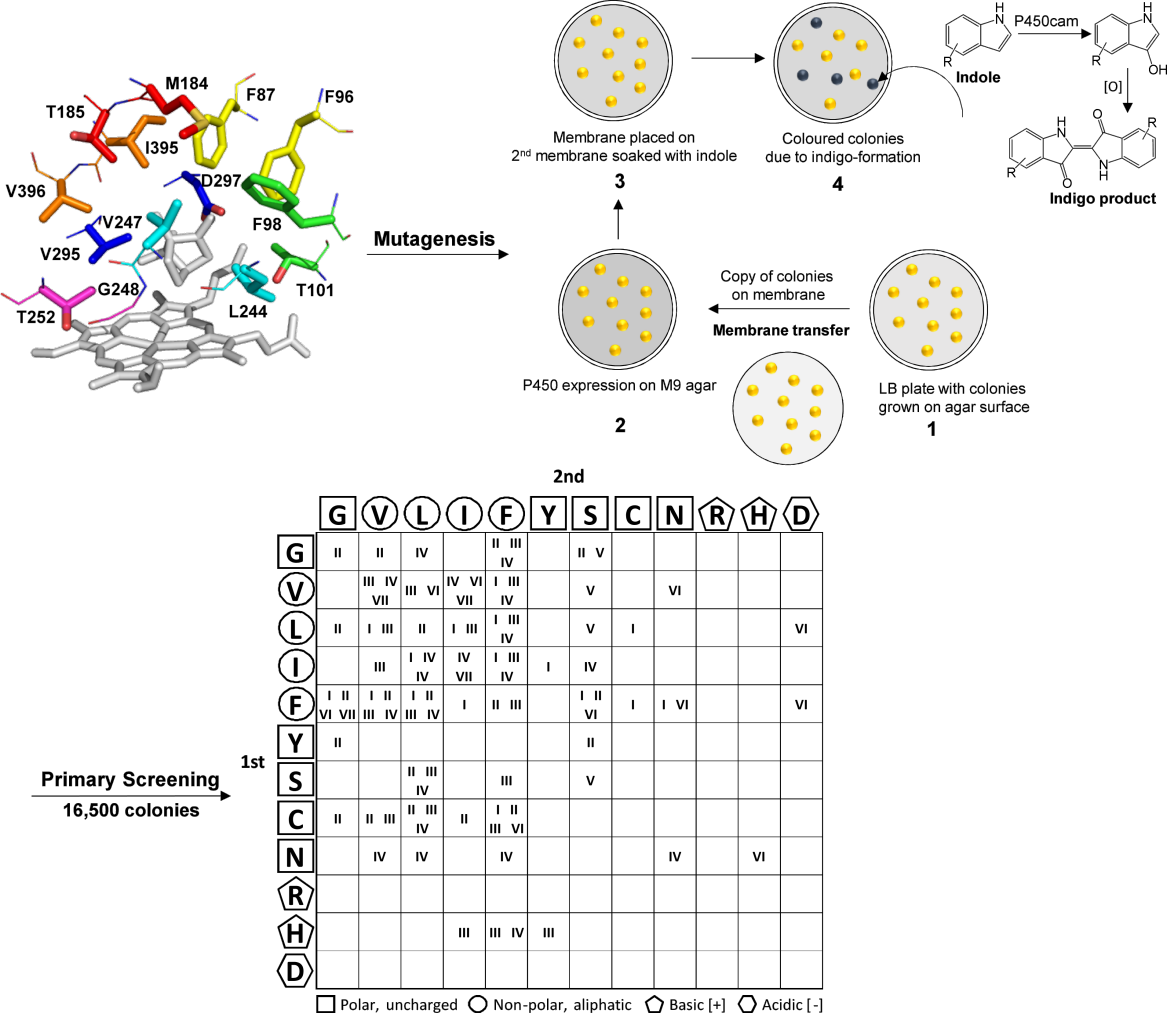
Library generation of P450cam[Tyr96Phe]-RhFRed. Active site of the P450cam-RhFRed variant Tyr96Phe (PDB ID: 1PHG) [26] with the 7 amino acid residue pairs representing the libraries Phe87/Phe96 I (yellow), Phe98/Thr101 II (green), Met184/Thr185 III (red), Leu244/Val247 IV (cyan), Gly248/Thr252 V (magenta), Val295/Asp297 VI (blue), Ile395/Val396 VII (orange). Following pairwise mutagenesis and solid-phase screening (using indole (**1**) as substrate), 93 new indigo positive variants were identified as represented in the grid. The grid represents all variant combinations identified across libraries I–VII. Rows in position 1 and columns in position 2 show the NDT amino acids classified according to their symbols in structure and chemical properties. The roman letters I–VII confirm that a member of that library with the amino acid configuration in position 1 and 2 has been found as an active enzyme – for example the mutant 98Gly/101Gly in library II (Phe98/Thr101) was found to be active as indicated in the top left box of the grid.

The P450cam-RhFRed libraries were generated by targeting 12 active site residues earlier specified by Loida and Sligar [21], which have been recently identified as universal selectivity determining positions within the P450 enzyme family [22]. In addition, Phe98 and Met184 mutant libraries were generated since Phe98 is thought to contribute to substrate orientation via hydrophobic interactions [23], whereas Met184 is part of the P450cam substrate recognition site 2 (SRS 2) [24]. Accordingly, the entire P450cam active site was partitioned into seven residue pairs which were targeted in site-directed mutagenesis experiments in the manner of CASTing (Figure 1) [25]. NDT codon degeneracy was introduced for each pair in turn, thus generating seven libraries I–VII as shown in the grid of Figure 1: Phe87/Phe96 (I), Phe98/Thr101 (II), Met184/Thr185 (III), Leu244/Val247 (IV), Gly248/Thr252 (V), Val295/Asp297 (VI) and Ile395/Val396 (VII).

Pairing of adjacent residues and the use of NDT codons helped to restrict the library size while still ensuring structural and functional diversity among the substituted residues. It was also hoped that favourable pairings would produce synergistic effects that might not otherwise have been discovered by substituting individual amino acids separately. Based on previous studies [10–11], the Tyr96Phe variant of P450cam was chosen as the template for screen development and subsequent mutagenesis. Thus, ≈16,500 colonies containing P450cam[Tyr96Phe]-RhFRed variants were rapidly screened for indigo formation (Figure 1, right), from which 93 new variants were identified in seven sub-libraries (Figure 1, bottom). Among this new population, structural and functional diversity was evident as can be seen from the grid structure (Figure 1, bottom) representing all active variant combinations identified across libraries I–VII. Cysteine, asparagine and histidine were not among the active site residues of the parent (or wild type) enzyme but appeared in several of the new variants, thus introducing a thiol, polar or basic group where previously none existed. More bulky aromatic side chains in libraries I and II were often substituted for smaller side chains, including that of Gly, introducing space in the upper part of the active site and substrate entrance channel. The small glycine side chain was substituted at seven different positions including former Phe, Thr, Met, Leu and Asp residues. Library II also included a Gly–Gly double substitution. Threonines in libraries II and III were often substituted for Phe, Gly or aliphatic side chains. The OH group was also frequently preserved by substitution with Ser, which in library V was always the case. Thr252 (library V) is involved in a proton relay network that promotes O–O bond scission during catalysis [21,27–29]. The retention of an OH group at position 252 is consistent with this important catalytic function. Indigo positive variants in library III substituted Met184 for Cys but also six of the other eleven NDT residues. Aliphatic residues Val, Leu and Ile in libraries IV, VI and VII were often interchanged with each other or Phe, but also Cys, Asn and Ser. Of all the sub-libraries, the fewest variants (just 4) were identified in library VII (I395–V396), possibly indicating an important role in indole binding and orientation for this amino acid pair. The acidic Asp residue was not identified except where it had previously existed at Asp297. Although this residue forms a hydrogen bond with the heme-7-propionate [30–32] several other residues, including His, were evident at this position, indicating that this interaction was not crucial for activity. Adding the P450cam-RhFRed library parent Tyr96Phe to the pool of 93 new variants gave a total of 94 for further screening.

### Investigation of P450cam activity toward a panel of substituted indoles

To begin exploring the substrate range of this new population, library I (Phe87/Phe96) variants were tested with a small panel of substituted indoles **1**–**4**.

**Figure 2:**
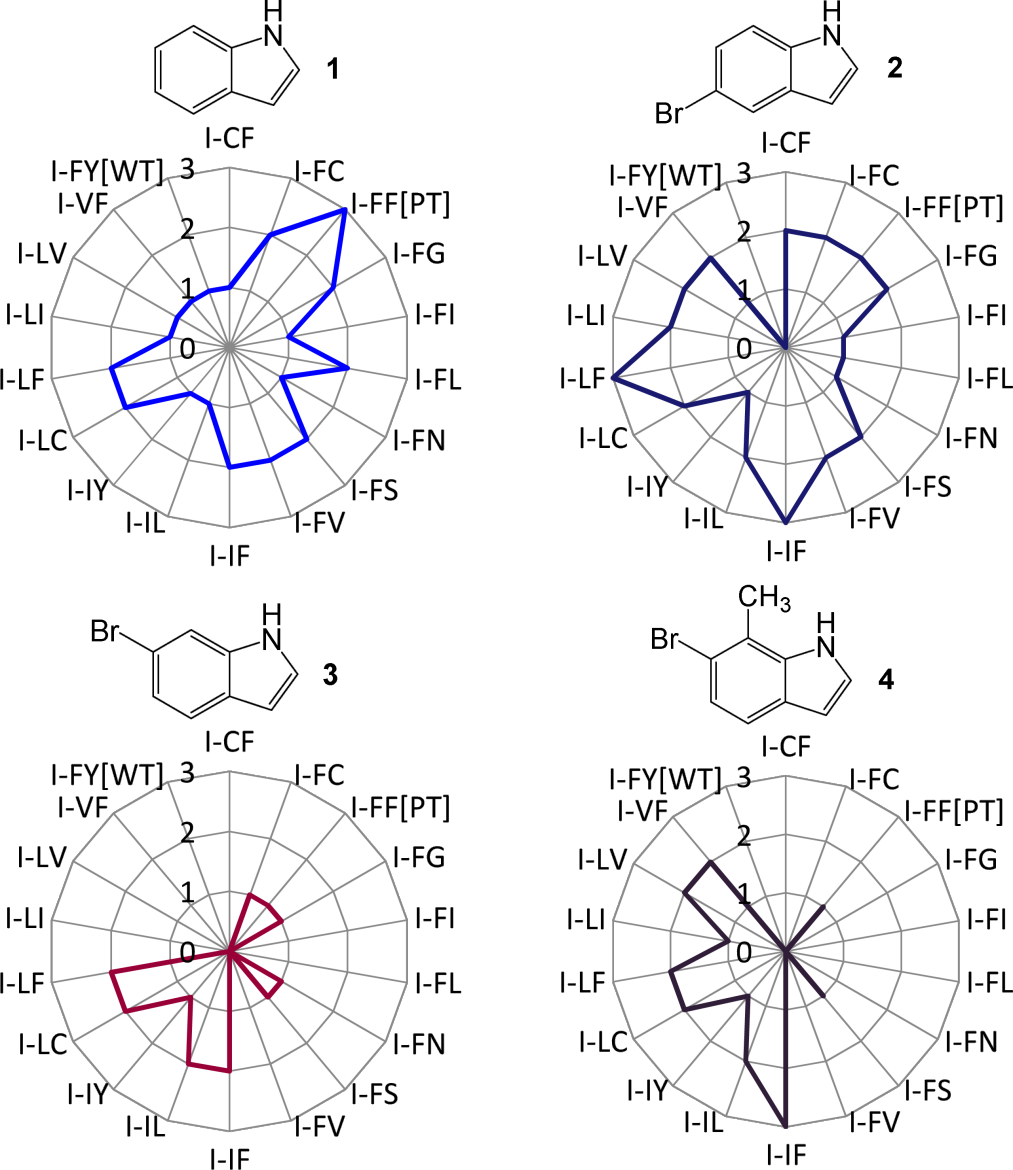
Radar plots illustrating the substrate acceptance of P450cam-RhFRed variants from library I. Colour formation in colonies was scored visually from 0–3, where 0 = no colour, 1 = low-level, 2 = mid-level and 3 = high-level colour. PT = parental type (P450cam[Tyr96Phe]-RhFRed), WT = wild type (P450cam-RhFRed).

Using a solid-phase screen as before, the level of colour formation in colonies was assessed visually, generating ‘fingerprints’ of activity as summarised in Figure 2 (also see Figure S1, Supporting Information File 1). The fingerprints show that variations in the configuration of the active site corresponded to variations in substrate acceptance. Variations in colour intensity might also be attributed to altered levels of active P450 or altered enzyme stability due to the substitutions made. If used in the context of an initial screen for activity following a diversification process, the use of indoles with P450s has a number of potential applications for enzyme optimisation studies and for developing protocols for neutral evolution. Selection for P450 variants that retain a threshold level of activity towards indole (**1**), such as the manner described herein, provides a diversified panel of variants with novel activities and increased capacity for improvement in subsequent rounds of directed evolution.

### Investigation of the P450cam libraries toward ethylbenzenes

To further explore the scope of variant libraries, the test substrate ethylbenzene (**5**), the *para*-methylated derivative **6** and the *para*-brominated derivative **7** were screened in liquid whole cell biotransformations (Tables S15–S21, Supporting Information File 1). Initially, indigo positive variants were combined from each library, with a roughly equal size of 5–8 variants per pool [33]. This pooling strategy allowed us to quickly identify active mutants without the need to screen all 93 variants separately. In addition, levels in P450 expression in library pools were assessed through CO difference spectroscopy [34] in order to distinguish between differences in activity due either to changes in specific activity or enzyme expression levels in the respective sub-pools (Tables S2–S8, Supporting Information File 1).

#### Screening for P450 expression

The levels in P450 expression were determined using CO difference spectroscopy in whole cells. The assay could be significantly improved both in terms of speed and safety by using carbon monoxide releasing molecules (CORMs) [35–37] as a source of CO rather than the gas CO itself. P450 concentrations determined in whole cells (1.1–5.9 µM) incubated with CORMs were similar or slightly higher when compared to concentrations determined in cell-free extracts (1.1–4.9 µM) treated with gaseous CO (Tables S2–S8, Supporting Information File 1). Based on this P450 quantification, very similar levels of expression were observed for all cells expressing the different P450 mutant pools (Table S9, Supporting Information File 1). Given the small differences in P450 expression observed, it was decided not to normalise enzyme activity to expression levels in subsequent activity studies.

#### Biotransformation reactions with library pools

**Figure 3:**
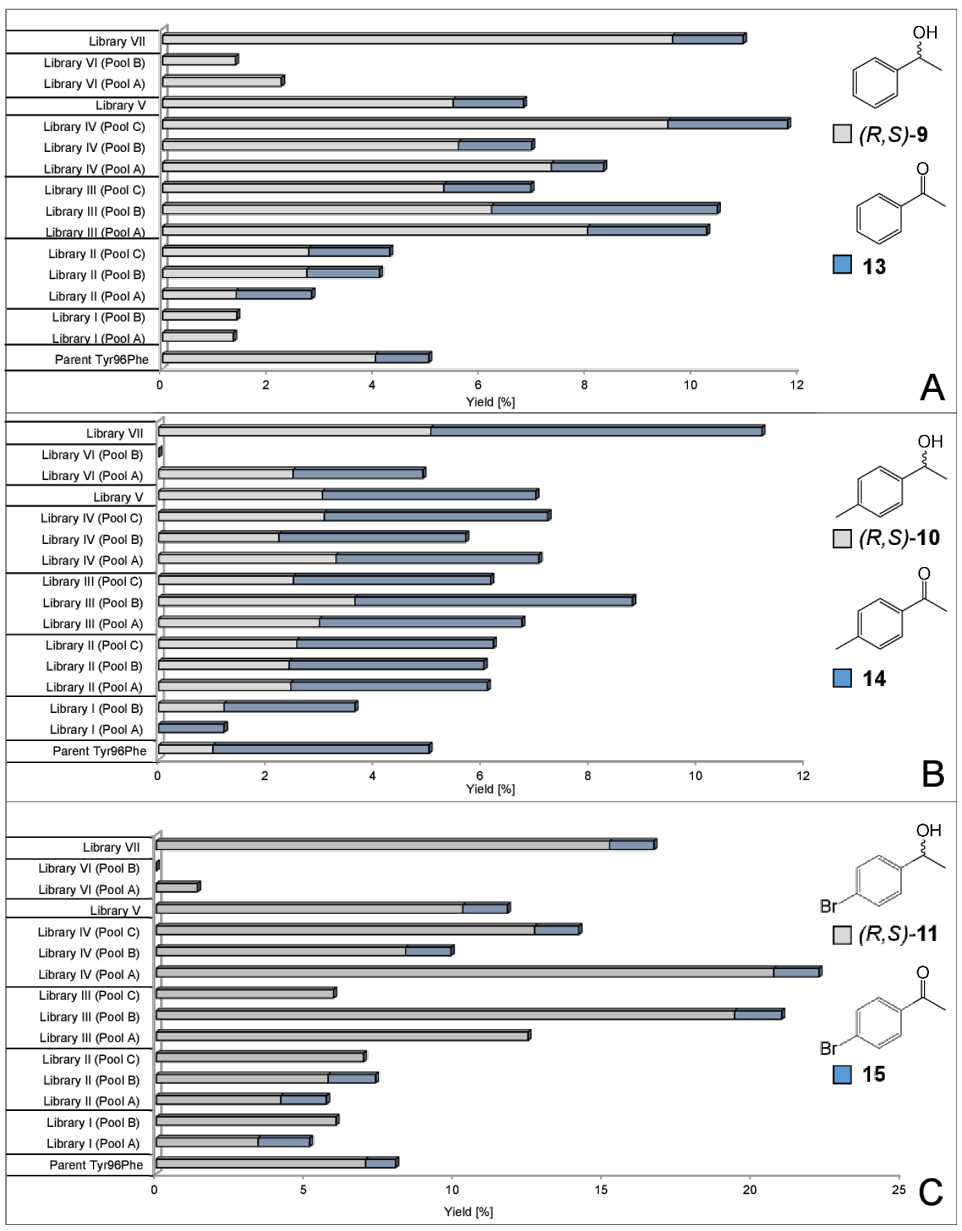
Yields of alcohols (*R*,*S*)-**9**-**11** (grey bars) and ketone products **13**–**15** (blue bars) in sub-pools of libraries I–VII and the Tyr96Phe parent with A) ethylbenzene (**5**), B) the *para*-methylated derivative **6** and C) the *para*-brominated derivative **7**. Reaction conditions: 180 mg/mL cells, 1 mM substrates, 50 mM sodium phosphate buffer (pH 7.2, 100 mM KCl, 0.4% glycerol (v/v)), 20 °C, 250 rpm, 48 h.

Biotransformations with pooled libraries and ethylbenzene (**5**) provided the average yield of alcohols (*R*,*S*)-**9** up to 10%, which is comparable to previously published data with isolated P450cam enzymes (Figure 3A) [21,27,38]. Highest concentrations of (*R*,*S*)-**9** were achieved in sub-pools of libraries III and IV revealing a 25–150% improvement in product formation as compared to the parent. With the *para*-methylated derivative **6** a pronounced over-oxidation to the ketone **14** occurred averagely yielding alcohols (*R*,*S*)-**10** in up to 5% with libraries I–VII showing distinct improvements in product yields when compared to the parental enzyme (Figure 3B). Library pools incubated with the *para*-brominated derivative **7** produced alcohols (*R*,*S*)-**11** in up to 21% as a significant improvement over the parent (Figure 3C). Overall yields were hampered by the volatility of starting materials, as shown by control experiments using dead cells, where 13% of starting material **5**, 17% of **6** and 19% of **7** were recovered (Table S22, Supporting Information File 1). Generally, the highest concentrations of (*R*,*S*)-alcohol products from compounds **5**–**7** were identified in sub-pools of libraries III (Met184/Thr185) and IV (Leu244/Val247) (Tables S17 and S18, Supporting Information File 1), which also seemed to contain the greatest diversity of variants.

#### Substrate specificity of individual library III variants toward ethylbenzene derivatives **5**–**8**

Following on from the results with pools of libraries I–VII, individual variants from library III (Met184/Thr185) were further investigated towards ethylbenzene derivatives **5**–**8** to see if chiral alcohols could be generated with improved rates compared to the parent variant Tyr96Phe (Table 1). Mutants harbouring the 185Phe mutation were specifically targeted since it was previously described that additional steric bulk at position 185 can lead to improved oxidation rates of ethylbenzene (**5**) [21,27].

**Table 1:** Product yields and ee’s obtained in biotransformation experiments with substrates **5**–**8** with the parent P450cam[Tyr96Phe]-RhFRed and indigo positive variants from library III (Met184/Thr185).^a^

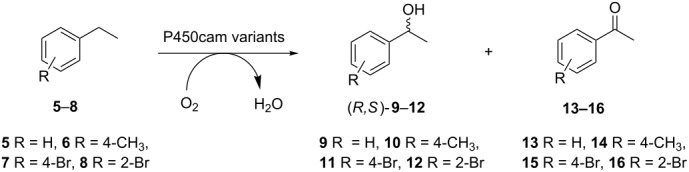				

Substrate	Variant	Overall yield [%] (*R,S*)-**9–12, 13–16**^b^	Yield [%] (*R,S*)-**9–12**	ee [%]^c^

**5**	Tyr96Phe	5	4	32 *(R)*
**5**	184Cys/185Phe	16	13	17 *(R)*
**6**	Tyr96Phe	5	4	35 *(S)*
**6**	184Cys/185Phe	6	5	9 *(S)*
**7**	Tyr96Phe	8	7	37 *(S)*
**7**	184Cys/185Phe	20	20	22 *(S)*
**8**	Tyr96Phe	16	11	6 *(S)*
**8**	184His/185Phe	46	37	15 *(S)*

^a^Reaction conditions: 2 mL scale, 180 mg/mL cells, 50 mM NaPi (pH 7.2, 0.4% glycerol (v/v), 100 mM KCl), 1 mM substrates **5**–**8**, 0.4% DMSO, 20 °C, 250 rpm, 48 h. ^b^Product yields determined by GC/FID. ^c^Enantioselectivities determined via chiral normal phase HPLC. All assays were accomplished in three replicates (Table S23, Supporting Information File 1).

The 184Cys/185Phe mutation produced a 2.5-fold improved formation of alcohols (*R*,*S*)-**9** with ethylbenzene (**5**) as compared to the parent Tyr96Phe albeit a decrease in ee from 32% (Tyr96Phe) to 17% (184Cys/185Phe) was evident. Interestingly, the *para*-methylated derivative **6** produced alcohol **10** with opposite (*S*)-selectivity both in the parent and mutant. Similar to substrate **6**, the *para*-brominated derivative **7** also produced (*S*)-selectivity with 2.4-fold improved yields of alcohol products (*R*,*S*)-**11** (20%) with the 184Cys/185Phe variant. In comparison to the *para*-bromo derivative **7**, the regioisomer **8** produced with the 184His/185Phe mutant significantly improved yields of (*R*,*S*)-**12** alcohols (37%) albeit with a slight decrease in selectivity (15%).

## Conclusion

A colony-based solid-phase screen for P450 indole activity was developed and used to generate a population of 93 indole active enzyme variants from screening a large library (16,500) of variants. The application of CORMs in place of the commonly used gaseous CO was found to be an attractive alternative for assessing P450 concentrations in whole cells. In a comprehensive pooling approach, P450cam mutants were shown to exhibit improved activities in the benzylic oxidation of ethylbenzene derivatives. The configuration of the newly generated chiral centre was highly dependent on substitution and subtle changes in substrate structure resulting in significant changes in both conversion and enantioselectivity. The active site library of 93 P450cam variants promises to be a useful tool for the discovery of new P450 activities and can be used as a starting point for further mutagenic studies.

## Supporting Information

File 1 General experimental information and procedures.
