# Supplementary material for: Active site diversification of P450cam with indole generates catalysts for benzylic oxidation reactions
Source: Beilstein J Org Chem. 2015 Sep 22;11:1713–20. doi: 10.3762/bjoc.11.186 (PMC4660908; doi:10.3762/bjoc.11.186)
Supplement: File 1 — General experimental information and procedures. [file Beilstein_J_Org_Chem-11-1713-s001.pdf]

Supporting Information

for

Active site diversification of P450cam with indole generates catalysts for benzylic oxidation reactions

Paul P. Kelly[‡1], Anja Eichler[‡1], Susanne Herter[1], David C. Kranz[1], Nicholas J. Turner[1] and Sabine L. Flitsch*[1]

Address: [1]School of Chemistry & Manchester Institute of Biotechnology, The University of Manchester, 131 Princess Street, M1 7DN, Manchester, United Kingdom

* Corresponding author

Email: Sabine L. Flitsch*– sabine.flitsch@manchester.ac.uk

‡ These authors contributed equally to the work.

Table of Contents

1 General experimental information and materials S3

1.1 Chemicals and consumables S3

1.2 General analytical procedures S3

1.3 Compounds investigated in colony-based solid-phase screens and biotransformation reactions S3

1.4 Strains, plasmids and media S4

1.5 PCR primers and conditions for library generation S4

1.6 Solid-phase screening with indole S5

1.7 Pooling of indigo-positive variants for secondary screening S7

2 Recombinant expression of P40camRhF[Tyr96Phe]-Red variants S7

2.1 Expression of single variants S7

2.2 Expression pooled variants S7

3 Determination of P450 concentrations S7

3.1 Whole cells S7

3.2 Cell free extracts S8

4 Biotransformations S12

4.1 General procedure for biotransformations S12

4.2 Biotransformations with pooled libraries S14

4.3 Biotransformations with single mutants S18

5 References S18

1 General experimental information and materials

1.1 Chemicals and consumables

All chemicals were of highest purity and obtained from Sigma-Aldrich (Poole, Dorset, UK) and Alfa Aesar (Karlsruhe, Germany) unless stated otherwise. GC/FID gases were purchased from BOC gases (Guildford, UK). Carbon monoxide was sourced from Sigma-Aldrich (Poole, Dorset, UK). Starting materials, authentic standards and reagents were purchased from Alfa Aesar (Karlsruhe, Germany) and Sigma-Aldrich (Poole, Dorset, UK) and used as received. Solvents were of analytical or HPLC grade and were purchased dried over molecular sieves where necessary. Amersham Hybond-N nitrocellulose membranes for solid phase screening were obtained from GE Healthcare (GE).

1.2 General analytical procedures

Solvents were of analytical or HPLC grade and dried over molecular sieves where necessary. Chiral normal phase HPLC was performed on an Agilent system (Santa Clara, CA, USA) equipped with a G1379A degasser, G1312A binary pump, a G1329 well plate autosampler unit, a G1315B diode array detector and a G1316A temperature controlled column compartment. The column used was a CHIRALCEL® OD-H column (5 µm particle size, 4.6 mm diameter × 250 mm; Daicel, Osaka, Japan). The typical injection volume was 10 µL and chromatograms were monitored at 210 nm. Conditions are indicated separately for each compound. GC analysis was performed on an Agilent 6850 GC (Agilent, Santa Clara, CA, USA) with a flame ionization detector (FID) and autosampler equipped with a 25 m Agilent CP-Chirasil-Dex CB column with a 0.6 mm inner diameter and 0.36 µm film thickness or with a 30 m Agilent J&W DB-1701 column with a 0.25 mm inner diameter and 0.25 µm film thickness (both from Agilent, Santa Clara, CA, USA).
Determination of P450 concentrations in whole cells was performed on a plate reader (Tecan Infinite 200 series, Männedorf, CH) and for cell-free extracts on a Cary 50 UV–visible spectrophotometer (Agilent Technologies, Santa Clara, CA, USA).

1.3 Compounds investigated in colony-based solid-phase screens and biotransformation reactions

Indole derivatives **1–4** were applied in solid-phase screenings. Ethylbenzene derivatives **5–8** were used in whole-cell biotransformation experiments.

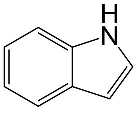

1

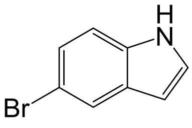

2

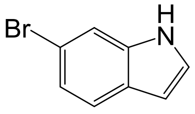

3

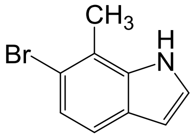

4

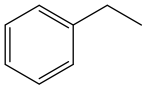

5

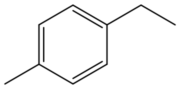

6

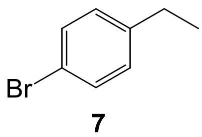

7

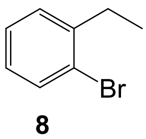

8

1.4 Strains, plasmids and media

The gene for the Tyr96Phe variant of P450cam, fused to RhF reductase, was cloned in a pET-14b expression vector (Novagen) as previously described [1,2]. The expression strain *E. coli* BL21 (DE3) was purchased from Merck4Biosciences UK as frozen stock of competent cells. XL1 Blue Supercompetent cells and XL10 Gold Ultracompetent cells were obtained from Stratagene (Agilent Technologies). High transformation efficiency of *E. coli* BL21 (DE3) cells were also purchased from Invitrogen (Carlsbad, CA). Cells were transformed using heat shock according to the manufacturer's instructions. Standard LB broth and LB agar plates were supplemented with 1% (w/v) glucose and 100 µg/mL ampicillin [3,4]. Liquid minimal medium and agar plates for expression contained 1X M9 minimal salts base [5] (Formedium, UK), 0.1 mM $CaCl_2$, 2 mM $MgSO_4$, 0.05% (w/v) $FeCl_3$, 0.4% (w/v) glucose and 100 µg/mL ampicillin.

1.5 PCR primers and conditions for library generation

Primers for the generation of active site mutants were designed as shown in Table S1. These include primers for the reversion of the Try96Phe mutation back to tyrosine and also the paired libraries I to VII. Primers (Eurofins Genomics, Ebersberg, GE) for the amplification of the P450cam-RhFRed gene are also given.

Table S1. Primers used for library generation.

Primer Name	Sequence (5' to 3')[a]
Cam_Phe96Tyr_Fwd	GAAGCCGGCGAAGCCTACGACTTCATTCCCACC
Cam_Phe96Tyr_Rev	GGTGGGAATGAAGTCGTAGGCTTCGCCGGCTTC
Lib-I_Phe87-Phe96_Fwd	CGAGTGCCCGNDTATCCCTAGAGAAGCCGGCGAAGCCNDTGACTTCATTC
Lib-I_Phe87-Phe96_Rev	GAATGAAGTCAHNGGCTTCGCCGGCTTCTCTAGGGATAHNCGGGCACTCG
Lib-II_Phe98-Thr101_Fwd	CGAAGCCTTTGACNDTATTCCCNDTTCGATGGATCCG
Lib-II_Phe98-Thr101_Rev	CGGATCCATCGAAHNGGGAATAHNGTCAAAGGCTTCG
Lib-III_Met184-Thr185_Fwd	CCTAACGGATCAGNDTNDTCGTCCAGATGGCAGCATGACC
Lib-III_Met184-Thr185_Rev	GGTCATGCTGCCATCTGGACGAHNAHNCTGATCCGTTAGG
Lib-IV_Leu244-Val246_Fwd	CAAGAGGATGTGTGGTNDTTTACTGNDTGGCGGCCTGGATACG
Lib-IV_Leu244-Val246_Rev	CGTATCCAGGCCGCCAHNCAGTAAAHNACCACACATCCTCTTG
Lib-V_Gly248-Thr252_Fwd	CCTGTTACTGGTCNDTGGCCTGGATNDTGTGGTCAATTTCCTC
Lib-V_Gly248-Thr252_Rev	GAGGAAATTGACCACAHNATCCAGGCCAHNGACCAGTAACAGG
Lib-VI_Val295-Asn297_Fwd	GCTTCTCGCTGNDTGCCNDTGGCCGCATCCTCACC
Lib-VI_Val295-Asn297_Rev	GGTGAGGATGCGGCCAHNGGCAHNCAGCGAGAAGC
Lib-VII_Ile395-Val396_Fwd	CACAAGAGCGGCNDTNDTAGCGGCGTGCAGGCA

Lib-VII_Ile395-Val396_Rev	TGCCTGCACGCCGCTAHNAHNGCCGCTCTTGTG
Cam_NcoI_Fwd	CATAGCCATGGGCACTGAAACCATACAAAG
Cam_BclI_Rev	CTAATGATCATTCAGAGTCGCAGGGCCAG

[a] Bases marked in red are deviations from the parental template sequence.

All PCRs were carried out using an Eppendorf Mastercycler Gradient thermal cycler (Hauppauge, NY). Mutagenic PCRs were conducted according to the QuikChange protocol (Agilent Technologies) with the QuikChange Lightning Site-Directed Mutagenesis Kit. Approximately 75 ng of P450cam[Tyr96Phe]-RhFRed (pET-14b) plasmid template and 125 ng of each primer was used in the PCR reactions. Initial denaturation at 95 °C for 2 min was followed by 18 cycles of denaturation at 95 °C for 20 s, annealing at 65 °C for 10 s and extension at 68 °C for 3 min 30 s. A final extension step of 68 °C for 5 min was also included.

Amplification of the P450cam gene from colonies was typically performed in a 25 µL PCR reaction by using 2 µL of colony solution (colonies were re-suspended in 50 µL of a 1:1 mixture of sterile Luria-Bertani medium (LB) [3,4] and dH_2O) as template and Pfu Ultra II Polymerase according to the manufacturer's instructions (New England Biolabs, Ipswich, MA, USA). Initial denaturation at 95 °C for 2 min was followed by 30 cycles of denaturation at 95 °C for 20 s, annealing at 65 °C for 20 sec and extension at 72 °C for 1 min 45 s. A final extension step of 72 °C for 5 min was also included.

Each of the seven libraries I to VII were generated separately by site-directed mutagenesis with NDT degenerate primers (Table S1). Following mutagenesis and transformation, all colonies were pooled for plasmid isolation. In order to minimise the amount of parental template in each plasmid library the mutagenesis procedure was generally performed twice, with the plasmid mixture from the first round acting as the template for the second round. After the second round, 10 to 20 colonies were picked and plasmids isolated for sequencing as a quality control for the mutagenesis procedure. The remaining colonies were collected and pooled for plasmid isolation.

1.6 Solid-phase screening with indole

For a primary screening, each of the plasmid libraries I to VII was transformed into *E. coli* BL21 (DE3). Transformants were spread on nitrocellulose membranes resting on LB agar plates (91 mm Petri dishes) [3,4]. After overnight incubation at 30 °C (until colonies were around 0.5–1.0 mm in diameter), membranes were transferred to M9 [5] expression plates. M9 expression plates were prepared by mixing the components as follows: 40% (w/v) glucose, 1 M $CaCl_2$, 1 M $MgSO_4$, 1000X trace metals [6] and 25% (w/v) $FeCl_3$, 1.5% (w/v) agar, 100 µg/mL ampicillin. On the surface of dried M9 expression plates 100 µL isopropyl β-D-1-thiogalactopyranoside (125 mM, IPTG) and 5-aminolevulinic acid (125 mM, 5-ALA) was spread. Colony-bearing membranes or cut out sections placed on M9 agar expression plates were left to incubate at 20 °C for around 20 h to induce protein expression. After protein expression, the membranes were transferred onto circles of gel blotting paper pre-soaked with substrate solution. The indole solution was prepared in 25 mL of buffer (50 mM sodium phosphate, pH 7.2, 100 mM KCl, 0.4% (v/v) glycerol) to a final concentration of 1 mM, with gentle heating to aid dissolution. The solution was poured over the blotting paper in a petri dish and the excess poured off. To develop colour the plates were incubated at room temperature for up to 24 h. Blue colonies were picked and typically used in a colony PCR.

The PCR products (the full-length P450cam-RhFRed gene with introduced mutations in the Cam domain) were then sequenced and re-cloned into pET-14b between the NcoI and BamHI sites as in the original construct. Re-cloned genes were expressed and the mutants re-screened with indole (**1**) as described in order to verify their activity. For each library around 10 'white' colonies were also selected for sequencing. In most cases the reads failed (presumably due to corrupted primer binding sites), were too short to give useful information or revealed large sections of mis-matching sequence. None of the 'white' sequences matched to the 'blue' sequences. In addition, library I (Phe87/Phe96) mutants were screened against a panel of substituted indole derivatives (**1–4**), shown in Figure S1.

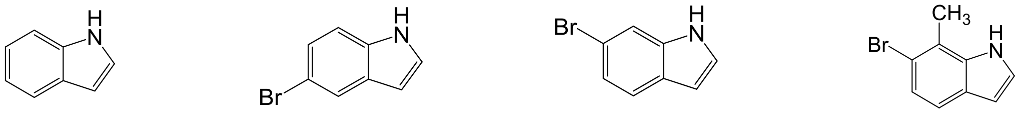

1 **2** **3** **4**

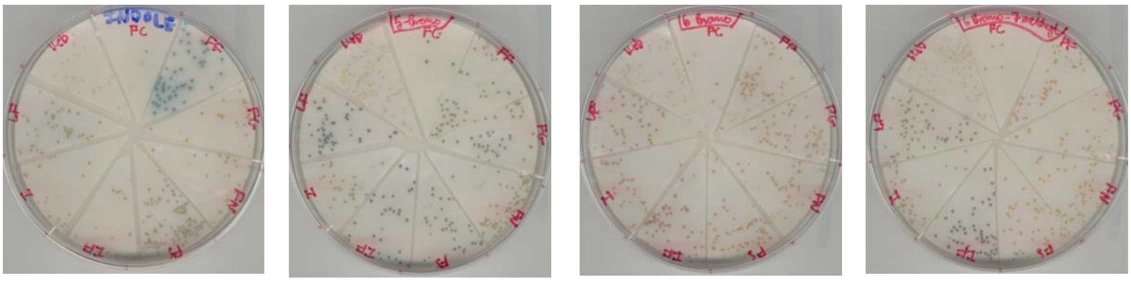

a

Position Indole (1) 5-Bromoindole (2) rate 6-Bromoindole (3) 6-Bromo-7-methylindole (4)

	87	96	Indole (1)	5-Bromoindole (2)	6-Bromoindole (3)	6-Bromo-7-methylindole (4)
	C	F	+	++	–	–
	F	C	++	++	+	–
[PT]	F	F	+++	++	+	+
	F	G	++	++	+	–
	F	I	+	+	–	–
	F	L	++	+	–	–
	F	N	+	+	+	–
	F	S	++	++	+	+
	F	V	++	++	–	–
	I	F	++	+++	++	+++
	I	L	+	++	++	++
	I	Y	+	+	+	+
	L	C	++	++	++	++
	L	F	++	+++	++	++
	L	I	+	++	–	+
	L	V	+	++	–	++
	V	F	+	++	–	++
[WT]	F	Y	+	–	–	–
b [14b]	–	–	–	–	–	–

Figure S1: Relative levels of colour formation from library I variants with substituted indoles. (a) Images of plates showing the colony colours evident from formation of the different substituted indigos. (b) The level of colour formation in colonies was assessed visually for the indigo-positive hits from library I, where "–" = no colour, "+" = low-level colour, "++" = mid-level colour and "+++" = high-level colour. [PT] denotes the parental type and [WT] the wild type. pET-14b was an empty vector control. Substrates used were (left to right) indole (**1**), 5-bromoindole (**2**), 6-bromoindole (**3**) and 6-bromo-7-methylindole (**4**).

.

1.7 Pooling of indigo-positive variants for secondary screening

The 94 new indigo positive variants identified from a colony-based screening were pooled to permit a secondary screening via biotransformation reactions. Expression of library variants in sub-pools was realised as follows: library I pool: 8 (A) + 8 (B) = 16 mutants, library II pool: 7 (A) + 7 (B) + 5 (C)= 19 mutants, library III pool: 8 (A) + 7 (B) + 6 (C) = 21 mutants, library IV pool: 7 (A) + 7 (B) + 5 (C) = 19 mutants, library V pool: 4 mutants, library VI pool: 5 (A) + 5 (B) = 10 mutants and library VII pool with 5 mutants. Plasmid pools were transformed into *E. coli* BL21 (DE3).

2 Recombinant expression of P450cam[Try96Phe]-RhFRed variants

2.1 Expression of single variants

For expression, the plasmid pET-14b containing the respective P450cam[Tyr96Phe]-RhFRed variant gene (or library variant gene) and the empty pET-14b plasmid was transformed into *E. coli* BL21 (DE3). A single colony was picked from an overnight LB plate and used to inoculate 10 mL LB medium [3,4] supplemented with ampicillin and grown overnight at 30 °C and 250 rpm. 5 mL of the overnight culture was used to inoculate 500 mL of supplemented M9 medium [5] in a 2-L-baffled Erlenmeyer flask. The cells were grown at 37 °C and 250 rpm to an optical density ($OD_{600\ nm}$) of 0.8–0.9. Subsequently, protein expression was induced by addition of IPTG (0.4 mM final concentration) and 5-ALA (0.5 mM final concentration). The cells were cultivated for protein expression at 20 °C and 250 rpm for 16 h. Cells were harvested by centrifugation at 4000 rpm and 4 °C for 20 min and washed once with 50 mM sodium phosphate buffer (pH 7.2, 0.4% glycerol (v/v), 100 mM KCl).

2.2 Expression of pooled variants

The procedure for expression of pooled variants (see section 2.1 above) followed that for single variants, except that an entire LB agar plate (of fresh transformants from overnight) of colonies was used to inoculate 10 mL LB medium. After incubation at 30 °C and 250 rpm, 5 mL of the overnight culture was used to inoculate 500 mL M9 [5] medium following the same expression protocol as described.

3 Determination of P450 concentrations

3.1 Whole cells

Cells were resuspended in buffer (1.3 M potassium phosphate, pH 8) to a concentration of 90 mg wet cells/mL. 180 µL of the cell suspension was transferred to a 96-well plate and 20 µL sodium dithionite (0.03 M) was added for reduction. The plate was subsequently mixed for 60 s before each scan. The reduced base-line spectrum was recorded between 400 to 500 nm in 1 nm intervals (Tecan Infinite 200 series, Männedorf, CH). After adding a few grains of CORM-3, the plate was mixed and scanned 5 to 6 times until the typical Fe^{II}-CO complex at 450 nm was identified [7-9]. All measurements were accomplished in triplicates.

3.2 Cell free extracts

Cell free extracts were prepared by dividing the resuspended cells (180 mg wet cells/mL in 50 mM NaPi, pH 7.2, 100 mM KCl and 0.4% glycerol (v/v)) in 10 mL portions and placing them on ice for cell disruption via ultrasonication (Soniprep 150 sonicator fitted with an exponential probe, MSE, London, UK). Cell lysates were centrifuged at 20,000 rpm for 15 min at 4°C. The P450 concentration of individual samples was determined by CO difference spectroscopy as described by Omura and Sato (1964) [10]. 1 mL of the resulting cell-free extract was transferred into a 1 mL cuvette, a few grains of sodium dithionite were added, the cuvette closed airtight with a septum and mixed by inversion. The baseline spectrum was recorded between a wavelength of 200 and 800 nm with 1 nm intervals using a dual-beam spectrophotometer (Cary 50 UV/Visible spectrophotometer, Agilent Technologies, Santa Clara, CA, USA). Afterwards, the airtight sample cuvette was aerated with 100% carbon monoxide for 30 s and the CO difference spectrum subsequently recorded. The P450 monooxygenase concentration was calculated using an extinction coefficient $\varepsilon_{450–490\ nm}$ of 91 $mM^{-1}cm^{-1}$ and was corrected to a path length of 0.55 (whole cell preparation) or 1 (cell-free preparation). All measurements were accomplished in three replicates (Tables S2–S9).

Table S2. Determination of P450 concentrations in whole cells and cell-free extracts of the P450cam-RhFRed library I (Phe87/Phe96) pool.

				Concentration [μM]	
	**Library pool**	**Mutant**	**Position**	**Whole cell**	**Cell free protein**
1	I (A)	Cys/Phe	87/96[a]	4.8 ± 1.23[b]	3.6 ± 0.29[c]
2	I (A)	Phe/Cys	87/96		
3	I (A)	Phe/Gly	87/96		
4	I (A)	Phe /Ile	87/96		
5	I (A)	Phe/Leu	87/96		
6	I (A)	Phe/Asn	87/96		
7	I (A)	Phe/Ser	87/96		
8	I (A)	Phe/Val	87/96		
9	I (B)	Ile/Phe	87/96	4.5 ± 0.12	4.1 ± 0.12
10	I (B)	Ile/Leu	87/96		
11	I (B)	Ile/Tyr	87/96		
12	I (B)	Leu/Cys	87/96		
13	I (B)	Leu/Phe	87/96		
14	I (B)	Leu/Ile	87/96		
15	I (B)	Leu/Val	87/96		
16	I (B)	Val/Phe	87/96		

[a] Phe87/Phe96 mutants bear the parental 96Phe mutation. [b] Conditions: 90 mg/mL cells, 0.03 M sodium dithionite and grains of CORM-3, 1.3M potassium phosphate buffer (pH 8). [c] Conditions: 180 mg/mL cells, grains of sodium dithionite followed by CO incubation, 50 mM sodium phosphate buffer (pH 7.2, 100 mM KCl, 0.4% glycerol (v/v)).

Table S3. Determination of P450 concentrations in whole cells and cell-free extracts of the P450cam-RhFRed library II (Phe98/Thr101) pool.

	Library pool	Mutant	Position	Concentration [μM] Whole cell	Concentration [μM] Cell free protein
1	II (A)	Cys/Phe	98/101[a]	4.9 ± 0.23[b]	3.8 ± 0.41[c]
2	II (A)	Cys/Gly	98/101		
3	II (A)	Cys/Ile	98/101		
4	II (A)	Cys/Leu	98/101		
5	II (A)	Cys/Val	98/101		
6	II (A)	Phe/Phe	98/101		
7	II (A)	Phe/Gly	98/101		
8	II (B)	Phe/Lys	98/101	3.3 ± 0.46	3 ± 0.2
9	II (B)	Phe/Ser	98/101		
10	II (B)	Phe/Val	98/101		
11	II (B)	Gly/Phe	98/101		
12	II (B)	Gly/Gly	98/101		
13	II (B)	Gly/Ser	98/101		
14	II (B)	Gly/Val	98/101		
15	II (C)	Leu/Gly	98/101	4 ± 2	4.9 ± 0.28
16	II (C)	Leu/Leu	98/101		
17	II (C)	Ser/Leu	98/101		
18	II (C)	Tyr/Gly	98/101		
19	II (C)	Tyr/Ser	98/101		

[a] Phe98/Thr101 mutants bear the parental 96Phe mutation. [b] Conditions: 90 mg/mL cells, 0.03 M sodium dithionite and grains of CORM-3, 1.3M potassium phosphate buffer (pH 8). [c] Conditions: 180 mg/mL cells, grains of sodium dithionite followed by CO incubation, 50 mM sodium phosphate buffer (pH 7.2, 100 mM KCl, 0.4% glycerol (v/v)).

Table S4. Determination of P450 concentrations in whole cells and cell-free extracts of the P450cam-RhFRed library III (Met184/Thr185) pool.

				Concentration [µM]	
	**Library pool**	**Mutant**	**Position**	**Whole cell**	**Cell free protein**
1	III (A)	Cys/Phe	184/185[a]	5.9 ± 0.62[b]	3.2 ± 0.08[c]
2	III (A)	Cys/Leu	184/185		
3	III (A)	Cys/Val	184/185		
4	III (A)	Phe/Phe	184/185		
5	III (A)	Phe/Leu	184/185		
6	III (A)	Phe/Val	184/185		
7	III (A)	Val/Phe	184/185		
8	III (A)	His/Phe	184/185		
9	III (B)	His/Ile	184/185	4.6 ± 0.61	3.2 ± 0.67
10	III (B)	Ile/Phe	184/185		
11	III (B)	Ile/Leu	184/185		
12	III (B)	Ile/Val	184/185		
13	III (B)	Leu/Phe	184/185		
14	III (B)	Leu/Ile	184/185		
15	III (B)	Leu/Leu	184/185		
16	III (C)	Leu/Val	184/185	2 ± 0.9	1.9 ± 0.03
17	III (C)	Ser/Phe	184/185		
18	III (C)	Ser/Leu	184/185		
19	III (C)	Gly/Phe	184/185		
20	III (C)	Val/Leu	184/185		
21	III (C)	Val/Val	184/185		

[a] Met184Thr185 mutants bear the parental 96Phe mutation. [b] Conditions: 90 mg/mL cells, 0.03 M sodium dithionite and grains of CORM-3, 1.3M potassium phosphate buffer (pH 8). [c] Conditions: 180 mg/mL cells, grains of sodium dithionite followed by CO incubation, 50 mM sodium phosphate buffer (pH 7.2, 100 mM KCl, 0.4% glycerol (v/v)).

Table S5. Determination of P450 concentrations in whole cells and cell-free extracts of the P450cam-RhFRed library IV (Leu244/Val247) pool.

	Library pool	Mutant	Position	Concentration [μM] Whole cell	Concentration [μM] Cell free protein
1	IV (A)	Cys/Leu	244/247[a]	1.7 ± 0.90[b]	2.60 ± 0.08[c]
2	IV (A)	Phe/Val	244/247		
3	IV (A)	Phe/Leu	244/247		
4	IV (A)	Gly/Phe	244/247		
5	IV (A)	Gly/Leu	244/247		
6	IV (A)	His/Phe	244/247		
7	IV (A)	Ile/Phe	244/247		
8	IV (B)	Ile/Ile	244/247	1.1 ± 0.19	2.3 ± 0.15
9	IV (B)	Ile/Leu	244/247		
10	IV (B)	Ile/Ser	244/247		
11	IV (B)	Leu/Phe	244/247		
12	IV (B)	Asn/Phe	244/247		
13	IV (B)	Asn/Leu	244/247		
14	IV (B)	Asn/Asn	244/247		
15	IV (C)	Asn/Val	244/247	4.5 ± 0.99	2.6 ± 0.10
16	IV (C)	Ser/Leu	244/247		
17	IV (C)	Val/Phe	244/247		
18	IV (C)	Val/Ile	244/247		
19	IV (C)	Val/Val	244/247		

[a] Leu244/Val247 mutants bear the parental 96Phe mutation. [b] Conditions: 90 mg/mL cells, 0.03 M sodium dithionite and grains of CORM-3, 1.3 M potassium phosphate buffer (pH 8). [c] Conditions: 180 mg/mL cells, grains of sodium dithionite followed by CO incubation, 50 mM sodium phosphate buffer (pH 7.2, 100 mM KCl, 0.4% glycerol (v/v)).

Table S6. Determination of P450 concentrations in whole cells and cell-free extracts of the P450cam-RhFRed library V (Gly248/Thr252) pool.

	Library pool	Mutant	Position	Concentration [μM] Whole cell	Concentration [μM] Cell free protein
1	V	Gly/Ser	248/252[a]	5.1 ± 1.56[b]	1.1 ± 0.01[c]
2	V	Lys/Ser	248/252		
3	V	Ser/Ser	248/252		
4	V	Val/Ser	248/252		

[a] Gly248/Thr252 mutants bear the parental 96Phe mutation. [b] Conditions: 90 mg/mL cells, 0.03 M sodium dithionite and grains of CORM-3, 1.3M potassium phosphate buffer (pH 8). [c] Conditions: 180 mg/mL cells, grains of sodium dithionite followed by CO incubation, 50 mM sodium phosphate buffer (pH 7.2, 100 mM KCl, 0.4% glycerol (v/v)).

**Table S7.** Determination of P450 concentrations in whole cells and cell-free extracts of the P450cam-RhFRed library VI (Val295/Asn297) pool.					
				**Concentration [µM]**	
	**Library pool**	**Mutant**	**Position**	**Whole cell**	**Cell free protein**
1	VI (A)	Cys/Phe	295/297[a]	4.4 ± 0.27[b]	4.9 ± 0.17[c]
2	VI (A)	Phe/Asn	295/297		
3	VI (A)	Phe/Gly	295/297		
4	VI (A)	Phe/Asn	295/297		
5	VI (A)	Phe/Ser	295/297		
6	VI (B)	Leu/Asn	295/297	2.8 ± 0.99	1.1 ± 0.09
7	VI (B)	Asn/His	295/297		
8	VI (B)	Val/Ile	295/297		
9	VI (B)	Val/Leu	295/297		
10	VI (B)	Val/Asn	295/297		

[a] Val295/Asn297 mutants bear the parental 96Phe mutation. [b] Conditions: 90 mg/mL cells, 0.03 M sodium dithionite and grains of CORM-3, 1.3M potassium phosphate buffer (pH 8). [c] Conditions: 180 mg/mL cells, grains of sodium dithionite followed by CO incubation, 50 mM sodium phosphate buffer (pH 7.2, 100 mM KCl, 0.4% glycerol (v/v)).

**Table S8.** Determination of P450 concentrations in whole cells and cell-free extracts of the P450cam-RhFRed library VII (Ile395/Val396) pool.					
				**Concentration [µM]**	
	**Library pool**	**Mutant**	**Position**	**Whole cell**	**Cell free protein**
1	VII	Phe/Gly	395/396[a]	5.8 ± 0.27[b]	2.3 ± 0.10[c]
2	VII	Ile/Ile	395/396		
3	VII	Val/Ile	395/396		
4	VII	Val/Val	395/396		

[a] Ile395/Val396 mutants bear the parental 96Phe mutation. [b] Conditions: 90 mg/mL cells, 0.03 M sodium dithionite and grains of CORM-3, 1.3M potassium phosphate buffer (pH 8). [c] Conditions: 180 mg/mL cells, grains of sodium dithionite followed by CO incubation, 50 mM sodium phosphate buffer (pH 7.2, 100 mM KCl, 0.4% glycerol (v/v)).

**Table S9.** Determination of P450 concentrations in individual mutants of library III (Met184/Thr185).				
			**Concentration [µM]**	
	**Mutant**	**Position**	**Whole cell**	**Cell free protein**
1	Phe (P_0)	96	5.6 ± 0.29[a]	2 ± 0.3[b]
2	Cys/Phe	184/185[c]	5.4 ± 0.25	3.1 ± 0.05
3	His/Phe	184/185	2.9 ± 0.67	2.4 ± 0.05

[a] Conditions: 90 mg/mL cells, 0.03 M sodium dithionite and grains of CORM-3,[5] 1.3M potassium phosphate buffer (pH 8). [b] Conditions: 180 mg/mL cells, grains of sodium dithionite followed by CO incubation, 50 mM sodium phosphate buffer (pH 7.2, 100 mM KCl, 0.4% glycerol (v/v)). [c] Met184/Thr185 mutants bear the parental 96Phe mutation.

4 Biotransformations

4.1 General procedure for biotransformations

Reactions were performed in sealed 7 mL glass vials in a 2 mL total reaction volume at 20 °C and 250 rpm. *E. coli* BL21 (DE3) cells expressing the respective variant enzyme were cultivated and harvested as described previously (see section 2.1) and resuspended in 50 mM sodium phosphate buffer (pH 7.2, 100 mM KCl, 0.4% glycerol (v/v)) to reach a final cell mass concentration of 180 mg/mL (wet cells). The substrates were dissolved in DMSO and

added to the cell suspension to a final concentration of 1 mM (0.4% (v/v) co-solvent). Samples (100 µL) were taken after 48 h and extracted into 500 µL MTBE. The percentual yield [%] was determined by GC/FID using the respective ketones of **13**–**15** or 2-heptanol as internal standard with conditions shown in Tables S10–S13. The enantiomeric excess (ee) was determined by normal phase HPLC using conditions shown in Table S14.

Table S10. GC/FID conditions used for analysis of product yields of biotransformation samples with compounds **5**–**7**.

Column	Agilent CP-Chirasil-Dex CB
Injector temperature	280°C
Oven temperature	100°C (1 min hold) - 120°C (10 min hold); rate 10°C/min; 12°C - 180°C (2 min hold);
Flow He [mL/min]	1.5
Pressure	15.5 bar (constant)
Detector temperature	250°C

Table S11. GC/FID conditions used for analysis of substrate conversion and product yields of biotransformation samples with compound **8**.

Column	Agilent J&W DB-1701
Injector temperature	200°C
Oven temperature	80°C (6.5 min hold) - 160°C; rate 10°C/min<br>160°C - 200°C; rate 20°C/min
Flow He [mL/min]	1.4
Pressure	14.4 bar (constant)
Detector temperature	200°C

Table S12. Retention times of starting materials **5**–**8**, (*R*,*S*)-alcohols **9-12** and ketones **13**–**16** obtained within GC/FID analysis.

Compound	Retention time [min]	Column
Ethylbenzene **5**	4.605	Agilent CP-Chirasil-Dex CB
(*R*)-1-Phenylethanol ***R*-9**	14.167	Agilent CP-Chirasil-Dex CB
(*S*)-1-Phenylethanol ***S*-9**	12.680	Agilent CP-Chirasil-Dex CB
1-Phenylethanone **13**	10.269	Agilent CP-Chirasil-Dex CB
1-Ethyl-4-methylbenzene **6**	5.839	Agilent CP-Chirasil-Dex CB
(*R*)-1-(4-Methylphenyl)ethanol ***R*-10**	20.968	Agilent CP-Chirasil-Dex CB
(*S*)-1-(4-Methylphenyl)ethanol ***S*-10**	19.045	Agilent CP-Chirasil-Dex CB
1-(4-Methylphenyl)ethanone **14**	15.301	Agilent CP-Chirasil-Dex CB
1-Bromo-4-ethylbenzene **7**	12.631	Agilent CP-Chirasil-Dex CB
(*R*)-1-(4-Bromophenyl)ethanol ***R*-11**	29.263	Agilent CP-Chirasil-Dex CB
(*S*)-1-(4-Bromophenyl)ethanol ***S*-11**	28.503	Agilent CP-Chirasil-Dex CB
1-(4-Bromophenyl)ethanone **15**	26.210	Agilent CP-Chirasil-Dex CB

**Table S13.** Retention times of starting materials and intermediates of biotransformation reactions with compounds **7** within GC-FID analysis.		
Compound	Retention time [min]	Column
2-Heptanol (internal standard)	7.493	Agilent J&W DB-1701
1-Bromo-4-ethylbenzene **7**	12.147	Agilent J&W DB-1701
1-(4-Bromophenyl)ethanol **(*R,S*-11)**	19.218	Agilent J&W DB-1701
1-(4-Bromophenyl)ethanone **15**	17.548	Agilent J&W DB-1701

**Table S14.** HPLC conditions and retention times of starting materials **5**–**8**, (*R*,*S*)-alcohols **9**–**12** and ketones **13**–**16**.				
Compound	Column	Solvent	Solvent ratio [%]	Retention time [min]
Ethylbenzene **5**	Chiracel® OD-H	Hexane:EtOH	98:2	3.642
(*R*)-1-Phenylethanol ***R*-9**	Chiracel® OD-H	Hexane:EtOH	98:2	8.174
(*S*)-1-Phenylethanol ***S*-9**	Chiracel® OD-H	Hexane:EtOH	98:2	9.554
1-Phenylethanone **13**	Chiracel® OD-H	Hexane:EtOH	98:2	5.379
1-Ethyl-4-methylbenzene **6**	Chiracel® OD-H	Hexane:EtOH	98:2	3.453
(*R*)-1-(4-Methylphenyl)ethanol ***R*-10**	Chiracel® OD-H	Hexane:EtOH	98:2	8.164
(*S*)-1-(4-Methylphenyl)ethanol ***S*-10**	Chiracel® OD-H	Hexane:EtOH	98:2	7.847
1-(4-Methylphenyl)ethanone **14**	Chiracel® OD-H	Hexane:EtOH	98:2	5.099
1-Bromo-4-ethylbenzene **7**	Chiracel® OD-H	Hexane:EtOH	98:2	3.617
(*R*)-1-(4-Bromophenyl)ethanol ***R*-11**	Chiracel® OD-H	Hexane:EtOH	98:2	9.291
(*S*)-1-(4-Bromophenyl)ethanol ***S*-11**	Chiracel® OD-H	Hexane:EtOH	98:2	8.704
1-(4-Bromophenyl)ethanone **15**	Chiracel® OD-H	Hexane:EtOH	98:2	5.837

4.2 Biotransformations with pooled libraries

Screening of substrates **5**–**7** with the pooled variants was originally conducted using whole cells at a concentration 180 mg/mL (wet weight) in 50 mM sodium phosphate (pH 7.2, 100 mM KCl, 0.4% glycerol (v/v)). 2 mL reactions containing 1 mM substrate from a 500 mM DMSO stock solution were incubated at 250 rpm and 20 °C in sealed 7 mL glass vials. 100 µL of samples were withdrawn after 48 h and extracted into MTBE (500 µL) for subsequent analysis by GC/FID (Tables S15–S21). To address the consumption of substrates due to their volatility, dead cells were prepared by autoclaving and used under the same conditions as described (Table S22). Each experiment was accomplished in three replicates.

**Table S15.** Product yields of chiral alcohols **9**–**11** and ketones **13**–**15** obtained from screening of P450cam-RhFRed library I (Phe87/Phe96) pools.[a]									
				**Yield [%] after 48 h**					
	**Pool**	**Mutant**	**Position**	**(*R*,*S*)-9, 13**	**(*R*,*S*)-9**	**(*R*,*S*)-10, 14**	**(*R*,*S*)-10**	**(*R*,*S*)-11, 15**	**(*R*,*S*)-11**
1	I (A)	Cys/Phe	87/96[b]	1.3 ± 0.03	<1[c]	1.2 ± 0.02	<1[c]	5 ± 0.6	3.4 ± 0.46
2	I (A)	Phe/Cys	87/96						
3	I (A)	Phe/Gly	87/96						
4	I (A)	Phe /Ile	87/96						
5	I (A)	Phe/Leu	87/96						
6	I (A)	Phe/Asn	87/96						
7	I (A)	Phe/Ser	87/96						
8	I (A)	PheVal	87/96						

9	I (B)	Ile/Phe	87/96	1.4 ± 0.07	<1[c]	2.4 ± 0.04	1.2 ± 0.01	6 ± 1	6 ± 1
10	I (B)	Ile/Leu	87/96						
11	I (B)	Ile/Tyr	87/96						
12	I (B)	Leu/Cys	87/96						
13	I (B)	Leu/Phe	87/96						
14	I (B)	Leu/Ile	87/96						
15	I (B)	Leu/Val	87/96						
16	I (B)	Val/Phe	87/96						

[a] Reaction conditions: 180 mg/mL cells, 1 mM substrates **9**–**11**, 50 mM sodium phosphate buffer (pH 7.2, 100 mM KCl, 0.4% glycerol (v/v), 20 °C, 250 rpm, 48 h). For analytical conditions see Tables S10 & S12. [b] Phe87/Phe96 mutants bear the parental 96Phe mutation. [c] Traces of (*R*,*S*)-alcohol and/or ketone detected.

Table S16. Product yields of chiral alcohols **9**–**11** and ketones **13**–**15** obtained from screening of P450cam-RhFRed library II (Phe98/Thr101) pools.[a]

				Yield [%] after 48 h					
	**Pool**	**Mutant**	**Position**	**(*R*,*S*)-9, 13**	**(*R*,*S*)-9**	**(*R*,*S*)-10, 14**	**(*R*,*S*)-10**	**(*R*,*S*)-11, 15**	**(*R*,*S*)-11**
1	II (A)	Cys/Phe	98/101[b]	2.8 ± 0.13	1.4 ± 0.05	3.7 ± 0.15	2.5 ± 0.08	6 ± 0.3	4 ± 0.3
2	II (A)	Cys/Gly	98/101						
3	II (A)	Cys/Ile	98/101						
4	II (A)	Cys/Leu	98/101						
5	II (A)	Cys/Val	98/101						
6	II (A)	Phe/Phe	98/101						
7	II (A)	Phe/Gly	98/101						
8	II (B)	Phe/Lys	98/101	3.7 ± 0.50	2.3 ± 0.49	3.7 ± 0.07	2.4 ± 0.05	7.3 ± 0.32	6 ± 0.1
9	II (B)	Phe/Ser	98/101						
10	II (B)	Phe/Val	98/101						
11	II (B)	Gly/Phe	98/101						
12	II (B)	Gly/Gly	98/101						
13	II (B)	Gly/Ser	98/101						
14	II (B)	Gly/Val	98/101						
15	II (C)	Leu/Gly	98/101	4.3 ± 0.36	2.7 ± 0.03	3.9 ± 0.33	2.6 ± 0.21	7 ± 0.5	7 ± 0.5
16	II (C)	Leu/Leu	98/101						
17	II (C)	Ser/Leu	98/101						
19	II (C)	Tyr/Gly	98/101						
18	II (C)	Tyr/Ser	98/101						

[a] Reaction conditions: 180 mg/mL cells, 1 mM substrates **9**–**11**, 50 mM sodium phosphate buffer (pH 7.2, 100 mM KCl, 0.4% glycerol (v/v), 20 °C, 250 rpm, 48 h). For analytical conditions see Tables S10 & S12. [b] Phe98/Thr101 mutants bear the parental 96Phe mutation.

**Table 17.** Product yields of chiral alcohols **9**–**11** and ketones **13**–**15** obtained from screening of P450cam-RhFRed library III (Met184/Thr185) pools.[a]									
				**Yield [%] after 48 h**					
	**Pool**	**Mutant**	**Position**	**(*R*,*S*)-9, 13**	**(*R*,*S*)-13**	**(*R*,*S*)-10, 14**	**(*R*,*S*)-10**	**(*R*,*S*)-11, 15**	**(*R*,*S*)-11**
1	III (A)	Cys/Phe	184/185[b]	10 ± 2	8 ± 2	4.2 ± 0.43	3 ± 0.4	12.4 ± 2.54	12.4 ± 2.54
2	III (A)	Cys/Leu	184/185						
3	III (A)	Cys/Val	184/185						
4	III (A)	Phe/Phe	184/185						
5	III (A)	Phe/Leu	184/185						
6	III (A)	Phe/Val	184/185						
7	III (A)	Val/Phe	184/185						
8	III (A)	His/Phe	184/185						
9	III (B)	His/Ile	184/185	10.4 ± 1.48	6.2 ± 0.28	5.2 ± 0.52	3.6 ± 0.06	21 ± 3	19 ± 3
10	III (B)	Ile/Phe	184/185						
11	III (B)	Ile/Leu	184/185						
12	III (B)	Ile/Val	184/185						
13	III (B)	Leu/Phe	184/185						
14	III (B)	Leu/Ile	184/185						
15	III (B)	Leu/Leu	184/185						
16	III (C)	Leu/Val	184/185	6.9 ± 0.50	5.3 ± 0.16	3.7 ± 0.09	2.5 ± 0.06	6 ± 0.5	6 ± 0.5
17	III (C)	Ser/Phe	184/185						
18	III (C)	Ser/Leu	184/185						
19	III (C)	Val/Phe	184/185						
20	III (C)	Val/Leu	184/185						
21	III (C)	Val/Val	184/185						
19	III (C)	Cys/Phe	184/185[b]						

[a] Reaction conditions: 180 mg/mL cells, 1 mM substrates **9–11**, 50 mM sodium phosphate buffer (pH 7.2, 100 mM KCl, 0.4% glycerol (v/v), 20°C, 250 rpm, 48 h). For analytical conditions see Tables S10 *&* S12. [b] Met184/Thr185 mutants bear the parental 96Phe mutation. [c] Traces of (*R*,*S*)-alcohol and ketone detected.

**Table S18.** Product yields of chiral alcohols **9**–**11** and ketones **13**–**15** obtained from screening of the P450cam-RhFRed library IV (Leu244/Val247) pools.[a]									
				**Yield [%] after 48 h**					
	**Pool**	**Mutant**	**Position**	**(*R*,*S*)-9, 13**	**(*R*,*S*)-13**	**(*R*,*S*)-10, 14**	**(*R*,*S*)-10**	**(*R*,*S*)-11, 15**	**(*R*,*S*)-11**
1	IV (A)	Cys/Leu	244/247[b]	8.3 ± 1.35	7.3 ± 0.50	4.7 ± 1.13	3.3 ± 0.96	22 ± 2	21 ± 2
2	IV (A)	Phe/Val	244/247						
3	IV (A)	Phe/Leu	244/247						
4	IV (A)	Gly/Phe	244/247						
5	IV (A)	Gly/Leu	244/247						
6	IV (A)	His/Phe	244/247						
7	IV (A)	Ile/Phe	244/247						
8	IV (B)	Ile/Ile	244/247	6.9 ± 0.50	5.6 ± 0.48	3.5 ± 0.11	2.2 ± 0.03	10 ± 0.7	8 ± 0.7
9	IV (B)	Ile/Leu	244/247						
10	IV (B)	Ile/Ser	244/247						
11	IV (B)	Leu/Phe	244/247						
12	IV (B)	Asn/Phe	244/247						
13	IV (B)	Asn/Leu	244/247						
14	IV (B)	Asn/Asn	244/247						

15	IV (C)	Asn/Val	244/247	11.8 ± 0.37	9.5 ± 0.32	4.3 ± 0.24	3.1 ± 0.21	14 ± 2	13 ± 2
16	IV (C)	Ser/Leu	244/247						
17	IV (C)	Val/Phe	244/247						
18	IV (C)	Val/Ile	244/247						
19	IV (C)	Val/Val	244/247						

[a] Reaction conditions: 180 mg/mL cells, 1 mM substrates **9**–**11**, 50 mM sodium phosphate buffer (pH 7.2, 100 mM KCl, 0.4% glycerol (v/v), 20 °C, 250 rpm, 4 8h). For analytical conditions see Tables S10 *&* S12. [b] Leu244/Val247 mutants bear the parental 96Phe mutation.

Table S19. Product yields of chiral alcohols **9**–**11** and ketones **13**–**15** obtained from screening of the P450cam-RhFRed library V (Gly248/Thr252) pool.[a]

	Pool	Mutant	Position	Yield [%] after 48 h					
				(*R*,*S*)-9, 13	(*R*,*S*)-13	(*R*,*S*)-10, 14	(*R*,*S*)-10	(*R*,*S*)-11, 15	(*R*,*S*)-11
1	V	Gly/Ser	248/252[b]	6.8 ± 1.05	5.5 ± 1.04	4.3 ± 0.37	3 ± 0.3	12 ± 0.4	10 ± 0.4
2	V	Lys/Ser	248/252						
3	V	Ser/Ser	248/252						
4	V	Val/Ser	248/252						

[a] Reaction conditions: 180 mg/mL cells, 1 mM substrates **9**–**11**, 50 mM sodium phosphate buffer (pH 7.2, 100 mM KCl, 0.4% glycerol (v/v), 20 °C, 250 rpm, 48 h). For analytical conditions see Tables S10 *&* S12. [b] Gly248/Thr252 mutants bear the parental 96Phe mutation.

Table S20. Product yields of chiral alcohols **9**–**11** and ketones **13**–**15** obtained from screening of the P450cam-RhFRed library VI (Val295/Asn297) pool.[a]

	Pool	Mutant	Position	Yield [%] after 48 h					
				(*R*,*S*)-9, 13	(*R*,*S*)-13	(*R*,*S*)-10, 14	(*R*,*S*)-10	(*R*,*S*)-11, 15	(*R*,*S*)-11
1	VI (A)	Cys/Phe	295/297[b]	2.2 ± 0.24	<1[c]	2.5 ± 0.08	<1[c]	3.4 ± 0.46	3.4 ± 0.46
2	VI (A)	Phe/Asn	295/297						
3	VI (A)	Phe/Gly	295/297						
4	VI (A)	Phe/Asn	295/297						
5	VI (A)	Phe/Ser	295/297						
6	VI (B)	Leu/Asn	295/297	1.4 ± 0.06	<1[c]	<1[c]	<1[c]	<1[c]	<1[c]
7	VI (B)	Asn/His	295/297						
8	VI (B)	Val/Ile	295/297						
9	VI (B)	Val/Leu	295/297						
10	VI (B)	Val/Asn	295/297						

[a] Reaction conditions: 180 mg/mL cells, 1 mM substrates **9**–**11**, 50 mM sodium phosphate buffer (pH 7.2, 100 mM KCl, 0.4% glycerol (v/v), 20 °C, 250 rpm, 48 h). For analytical conditions see Tables S10 *&* S12. [b] Val295/Asn297 mutants bear the parental 96Phe mutation. [c] Traces of (*R*,*S*)-alcohol and/or ketone detected.

Table 21. Product yields of chiral alcohols **9**–**11** and ketone **13**–**15** obtained in screening the P450cam-RhFRed library VII (Ile395/Val396) pool.[a]

	Library pool	Mutant	Position	Yield [%] after 48 h					
				(*R*,*S*)-9, 13	(*R*,*S*)-13	(*R*,*S*)-10, 14	(*R*,*S*)-10	(*R*,*S*)-11, 15	(*R*,*S*)-11
1	VII	Phe/Gly	395/396[b]	10.9 ± 0.59	9.6 ± 0.59	6.4 ± 0.34	5 ± 0.3	17 ± 1	15 ± 1
2	VII	Ile/Ile	395/396						
3	VII	Val/Ile	395/396						
4	VII	Val/Val	395/396						

[a] Reaction conditions: 180 mg/mL cells, 1 mM substrates **9**–**11**, 50 mM sodium phosphate buffer (pH 7.2, 100 mM KCl, 0.4% glycerol (v/v), 20 °C, 250 rpm, 48 h). For analytical conditions see Tables S10 *&* S12. [b] Ile395/Val396 mutants bear the parental 96Phe mutation.

To determine the substrates volatility and adhesion to cells, biotransformation reactions with autoclaved cells (dead cells) were accomplished with substrates **5–7** and (*R*,*S*)-**9–11** alcohols (procedure followed according to section 4.1).

Table S22. Recovered starting materials **5**–**7** and (*R*,*S*)-**9**–**11** alcohols extracted from autoclaved cells after 48 h.[a]

Compound	Recovered starting material [%]
**5**	13 ± 0.3
(***R***)-**9**	4.1 ± 0.47
(***S***)-**9**	4 ± 0.4
**6**	17 ± 1
(***R***)-**10**	2.6 ± 0.31
(***S***)-**10**	2.5 ± 0.34
**7**	19.2 ± 0.56
(***R***)-**11**	8 ± 1
(***S***)-**11**	8 ± 1

[a] Reaction conditions: 180 mg/mL dead cells, 1 mM substrates **5**-**7** or (*R*,*S*)-**9**–**11**, 50 mM sodium phosphate buffer (pH 7.2, 100 mM KCl, 0.4% glycerol (v/v)) 20 °C, 250 rpm, 48 h. For analytical conditions see Tables S10 *&* S11.

4.3 Biotransformations with single mutants

The P450cam[Tyr96Phe]-RhFRed mutant plus mutants from library III (Met184/Thr185) were examined for their activity toward the substrate panel of ethylbenzenes (**5**–**8**) within whole cell biotransformation reactions (Table S23) as described previously (see section 4.1). Product yields were verified by GC/FID (in %, Tables S12 and S13) and the enantiomeric excess (ee in %) of the produced (*R*,*S*)-**9**-**12** alcohols were determined by normal phase HPLC (Table S14). Each experiment was accomplished in three replicates.

Table S23. Product yields and *e.e.*'s obtained in biotransformation reactions with the parent P450cam[Tyr96Phe]-RhFRed and library III (Met184/Thr185) mutants.[a]

Compound	Variant	Overall yield [%] (R,S)-9-12, 13-16	Overall yield [%] (R,S)-9-12	ee [%]
**5**	Tyr96Phe	5 ± 0.3	4 ± 0.3	32 ± 1 *(R)*
**5**	184Cys/185Phe	16 ± 0.7	13 ± 0.5	17 ± 0.1 *(R)*
**6**	Tyr96Phe	5 ± 0.2	4 ± 0.1	35 ± 1 *(S)*
**6**	184Cys/185Phe	6 ± 0.3	5 ± 0.2	9 ± 0.1 *(S)*
**7**	Tyr96Phe	8 ± 0.9	7 ± 0.8	37 ± 0.4 *(S)*
**7**	184Cys/185Phe	20 ± 1	20 ± 1	22 ± 0.7 *(S)*
**8**	Tyr96Phe	16 ± 2	11 ± 2	6 *(S)* [b]
**8**	184His/185Phe	46 ± 4	37 ± 0.7	15 *(S)* [b]

[a] Reaction conditions: 180 mg/mL dead cells, 1 mM substrates **5**–**8**, 50 mM sodium phosphate buffer (pH 7.2, 100 mM KCl, 0.4% glycerol (v/v) 20 °C, 250 rpm, 48h. For analytical conditions see Tables S10–S14. [b] *e.e.* value was calculated from a single measurement.

5 References

1. Robin, A.; Roberts, G. A.; Kisch, J.; Sabbadin, F.; Grogan, G.; Bruce, N.; Turner, N. J.; Flitsch, S. L. *Chem. Comm*. **2009,** 2478-2480.
2. Sabbadin, F.; Hyde, R.; Robin, A.; Hilgarth, E. M.; Deleune, M.; Flitsch, S.;Turner, N.; Grogan, G.; Bruce, N. C. *ChemBioChem*, **2010,** *11,* 987-994.

3. Bertani, G. *J. Bacteriol*. **1951,** *62,* 293-300.
4. Sambrook, J.; Russell, D. W. *Molecular Cloning: A Laboratory Manual*; Cold Spring Harbor Laboratory Press: Cold Spring Harbor, NY; 2001.
5. Maike, H. (Eds.: Davis, L. G.; Dibner, M. D.; Battey, J. F.) *Basic Methods in Molecular Biology*; Elsevier: London; Vol. 29, 1986, p. 336.
6. Studier, F. W. *Protein Expr. Purif*. **2005,** *41,* 207-234.
7. Geier, M.; Braun, A.; Emmerstorfer, A.; Pichler, H.; Glieder, A. *Biotechnol. J*. **2012,** *7,* 1346-1358.
8. Gudiminchi, R. K.; Geier, M.; Glieder, A.; Camattari, A. *Biotechnol. J*. **2013,** *8,* 146-152.
9. Garcia-Gallego, S.; Bernardes, G. J. L. *Angew. Chem. Int. Ed*. **2014,** *53,* 9712-9721.
10. Omura, T.; Sato, R. *J. Biol. Chem*. **1964,** 239*,* 2370-2378.
